# A Compact Fiber Inclinometer Using a Thin-Core Fiber with Incorporated an Air-Gap Microcavity Fiber Interferometer

**DOI:** 10.3390/s16010092

**Published:** 2016-01-12

**Authors:** Jiacheng Li, Xueguang Qiao, Qiangzhou Rong, An Sun

**Affiliations:** 1 Physics Department, Northwest University, No.229, Taibai Road (North), Xi’an 710069, China; jcli@stumail.nwu.edu.cn; 2 International Institute for Urban Systems Engineering, Southeast University, PailouFour No. Two, Nanjing 210096, China; sunan_1978@163.com

**Keywords:** Fabry-Perot interferometer, inclinometer, fringe contrast

## Abstract

A compact fiber-optic inclinometer is proposed and experimentally demonstrated based on a Fabry-Perot interference (FFPI). The sensing head consists of a short segment of thin-core fiber (TCF) following with a piece of hollow-core fiber (HCF). High-order cladding modes have been excited because of core diameter mismatch. A clear interference spectrum has been obtained as the consequence of interference among the reflected core modes and cladding modes. Fringe contrast of the interference spectrum is highly sensitive to fiber bending with direction independence, and good linearity has been observed during the bending range from 1° to 3° with a sensitivity of 2.71 dB/deg.

## 1. Introduction

Inclinometers (tilt sensors) are of great interest in the inaccurate measurement of objects’ angular variation. Fiber-optic inclinometers have been developed in various schemes, which can be mainly classified into two types: the grating-based and the interferometer-based [1,2,3,4,5]. The fiber Bragg grating (FBG) is inherently insensitive to micro-bending since the light is confined in the core area. A solution is to incorporate an etching segment of fiber that serves as a core-to-cladding mode coupler in the upstream of grating, and a sensitivity of 0.43 dBm/deg in bending measurement can be obtained [6]. This pre-processing enables FBG to be sensitive to bending, but weakens the mechanical strength of the sensor. Thanks to the core mismatch between the single mode fiber (SMF) and taper waist [7,8], a nonadiabatic taper can be also used for the coupling of core-to-cladding modes of FBG. For example, a tapered FBG-based inclinometer has been proposed for temperature-insensitive bending measurements with a large range from 0° to 90° [8]. A taper upstream-tilted FBG (TFBG) can recouple the cladding modes back to the leading-in fiber, making TFBG a probe-type bending sensor by interrogating the power of recoupled cladding modes. For example, a bending sensor has been proposed based on a TFBG, and the intensity of the resonance peaks is sensitive to the bending operations with a maximum sensitivity of 0.174 dB/m^−1^ [9]. Similarly, the combination of long period fiber grating (LPG) and fused tapers is also a typical structure to couple core modes to the cladding modes [10]. The interference between the forward-propagating core and cladding modes could be sensitive to the bending angle because of the bending-induced refractive index (RI) change of the taper region. These kinds of grating-based structures mentioned above are reliable and can be applied as tilt sensors, but generally involve the complex fabrication process of a taper section using a sophisticated splicer [10] or flame-fused tapering equipment [11], which are expensive and weaken the mechanical strength of the taper region. In addition, as another type of fiber-optic device, fiber interferometers with separate interference arms are also sensitive to bending. Especially, the in-fiber interferometer is of more interest in bending sensing because of its compactness. It is significant to induce different waves transmitting with phase delay difference in a single fiber based on diverse interference mechanisms. For example, the twin core fiber-based interferometers have been developed for highly sensitive bending sensing [12,13]. Another common technique is to employ splices between similar or dissimilar fibers with core-mode-field mismatching, in which the expected interference spectrum may be achieved via the interference between core and cladding modes [14,15,16,17,18]. For example, a hollow-core photonic crystal fiber (PCF) was spliced between two SMFs with a lateral offset to form an interferometer. Bending affects the multiple modes differently in RI and furthers the interference spectrum, making it possible to measure bend angles [18]. These devices can be used for bending angle measurement; however, its transmission-based type limits its application. On the other hand, the reflection-based components show unique advantages [19,20,21]. For example, one of our prior works proposed an introduction of a reflection-type interferometer formed by a polarization-maintaining photonic crystal fiber (PM-PCF) [19]. The interference among the polarized modes was capable of responding to bending with direction-dependence. As another typical reflection-type device, fiber Fabry-Perot interferometers (FFPI) have also attracted great interest owing to their advantages of high stability, compact size and simple fabrication. The FFPIs have been used to measure diverse physical parameters (e.g., temperature [22], pressure [23] and refractive index [24]) by interrogating the changes of the interference wavelength and fringe contrast. However, these FFPIs were based on the interference between the confined core modes and presented micro-structure size, which limits their application for the measurement of bending angles.

In this paper, we propose and experimentally demonstrate a compact FFPI interferometer-based inclinometer fabricated by simply self-aligned splicing. The sensing device is fabricated by incorporating a short segment of thin-core fiber (TCF) upstream of a FFPI. The interferometer is highly sensitive to fiber bending, which makes it a good candidate as an interferometer for tilt-angle measurement.

## 2. Operation Principle

**Figure 1:**
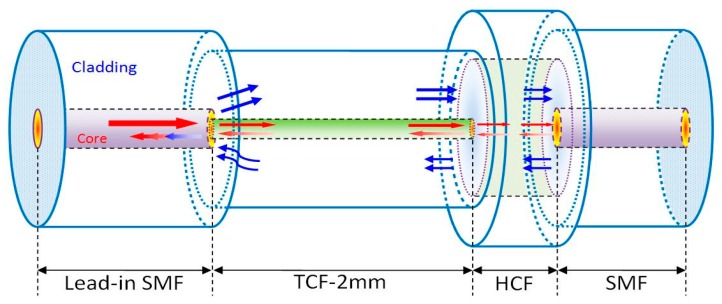
Schematic diagram of the FFPI structure.

The schematic diagram of the interferometer is shown in Figure 1. The sensing regions mainly composed of a 2 mm long TCF and a sandwiched structure of SMF-HCF (50 μm in length)-SMF, making an in-line air-gap micro-cavity FFPI. The different parts are self-aligned and spliced with each other at the discharge condition: +20 bit for power and 600 ms for time, with a fusion splicer (Fujikura, FSM-60S). The 50 μm long HCF can be obtained by precise cleaving under a microscope (Nikon ECLIPSE LV100) with a magnification of 15 times. Furthermore, the TCF and SMF is spliced with a self-aligned process which provides a constant discharge condition. Therefore, the two operations above are capable of determining the splicing between TCF and SMF repeatedly. The core and cladding diameters of the TCF are 4.0 µm and 80 µm, respectively. The length of the TCF is carefully selected through trials and errors to ensure good mode excitation, efficiency, and compactness. The diameters of the core and cladding of HCF are 30 μm and 150 μm, respectively. Considering that the end-face of the leading-out SMF can reflect the core mode and cladding modes and may influence the interference spectrum, two ways have been employed to prevent this “bad” reflection of leading-out the SMF’s end-face. One is that the leading-out SMF is freely cleaved, and thus the end-face is rough, which significantly decreases the reflectivity. The other way is using an ~10 cm long leading-out SMF as the pigtail fiber without stripping the coating, which can result in a large loss of cladding modes transmitting within the cladding of the SMF. Therefore, the reflection influence of the end-face is so slight that it can be ignored.

**Figure 2:**
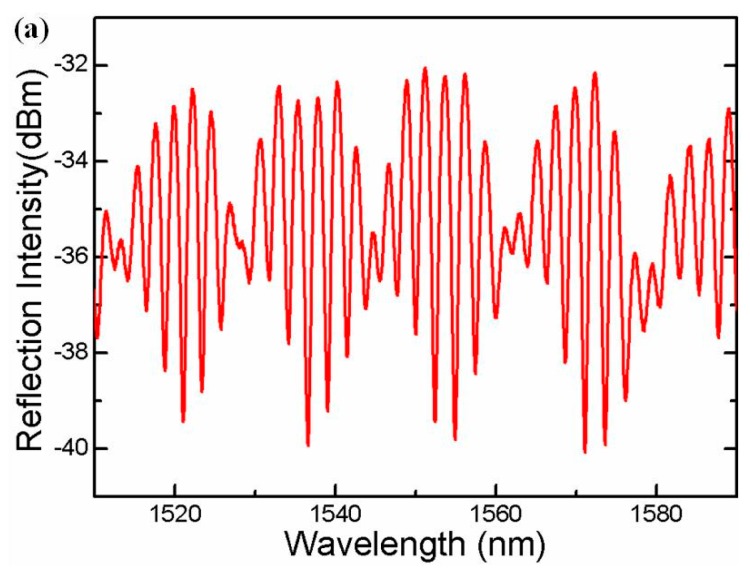

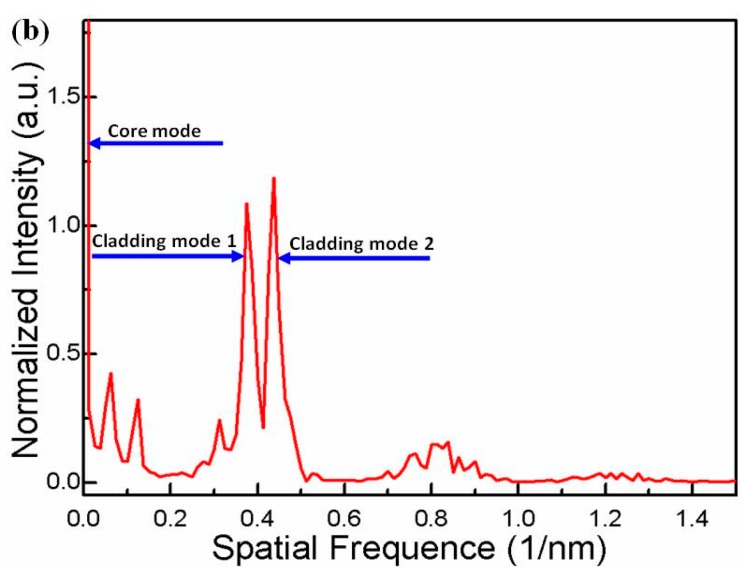
(**a**) Reflective spectrum of the FFPI configuration; (**b**) FFT spectrum of the reflective spectrum.

In this structure, part of the core-guided fundamental mode couples to the cladding modes because of the core diameter mismatching between the TCF and SMF. These cladding modes and the residual core mode transmit along the cladding and core of the TCF to the FP cavity. Parts of the light are reflected back to the SMF by two surfaces of the FP cavity (*i.e.*, two end-faces on both sides of HCF) and are finally recoupled to the leading-in SMF. Because of the phase delay, the interference occurs between reflection waves, resulting in a well-defined interference spectrum, as shown in Figure 2a. To further analyze the characteristics of the interference patterns, the wavelength spectra in Figure 2a aretransformed to the spatial frequency using the Fast Fourier Transform Algorithm, as shown in Figure 2b. It can be seen that the generation of the interference spectrum mainly consists of three mechanisms: interference between the reflection core mode, interference between the core mode and cladding modes, interference between the cladding modes. Therefore, the interference pattern does present inhomogeneity. Generally, cladding modes are easily disturbed by the deformation of the fiber because of its mode field distribution, especially in the TCF which is incapable of confining the cladding modes. The bending and deflection operations on the sensing area change the refractive index across the fiber, and thus affect the cladding modes participating in the interference spectrum in two ways: the coupling between the backward-propagating core mode and the forward-propagating cladding modes in the TCF, and the coupling between the core mode of the SMF and the cladding modes of the TCF. As a result, the fiber-bending decreases both kinds of couplings over the recoupled cladding modes, and has no influence on the core mode. The cladding modes’ change will sensitively influence the fringe contrast of the interference pattern. This character makes the proposed sensor have the ability of detecting the micro-displacement as the following demonstration in the experiment. In addition, the bending also induces the refractive indices’ change over the TCF and splicing region, as well as the length change of the sensing area. These two effects will change the phase differences among the recoupled core and cladding modes, resulting in the interference spectrum shift.

## 3. Experimental Results and Discussion

**Figure 3:**
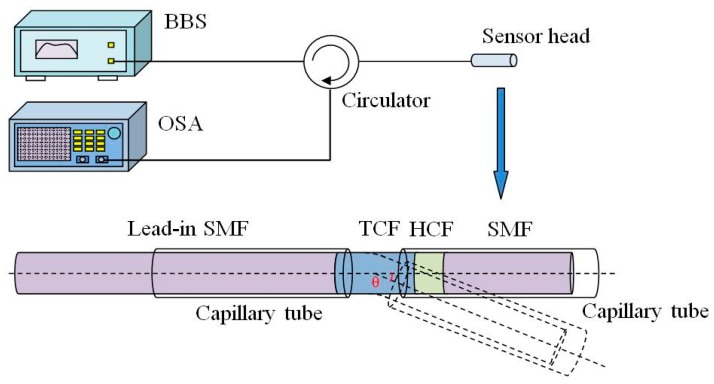
Schematic diagram of the experimental setup for bending angle measurement.

**Figure 4:**
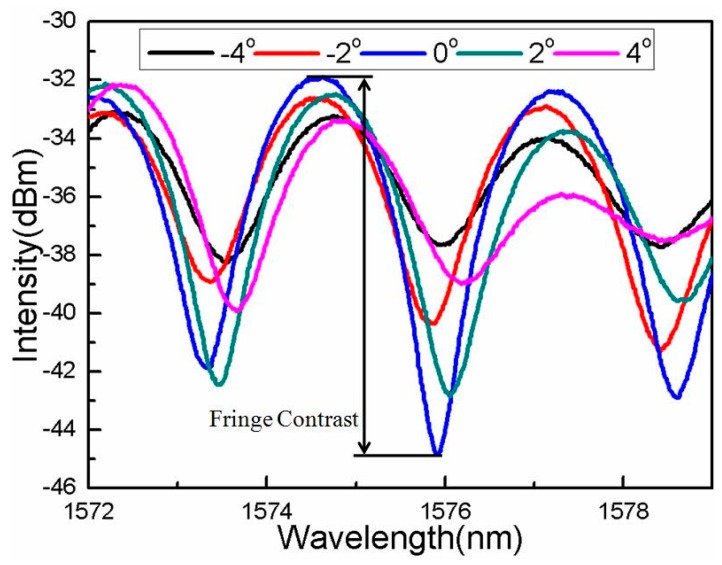
Interference spectral response to different bending angles.

Figure 3 shows the schematic diagram of the experimental setup. A broad band light source (BBS) (Hoyatek, ASE-C+L module) is used to illuminate the interferometer via a circulator. The reflection spectrum is observed by using an optical spectrum analyzer (OSA) (YOKOGAWA, AQ6370B), the wavelength resolution of which is 0.02 nm and the intensity resolution of which is 0.01 dB.In the experiment, in order to protect the fiber and keep it straight for any bending angle, two capillary tubes are used on both sides of the rotating axis, as shown in Figure 3. The bending area is the fiber between the two capillary tubes (1.5 mm). One of capillary tubes is fixed at a stage, while the other one is fixed on a rotator. The fringe contrast of the interference valley near 1576 nm is used as an indicator for the bending angle measurement. Once the bending is implemented on the interferometer, the fringe contrast sharply decreases with the increasing bending angle, as shown in Figure 4.

**Figure 5:**
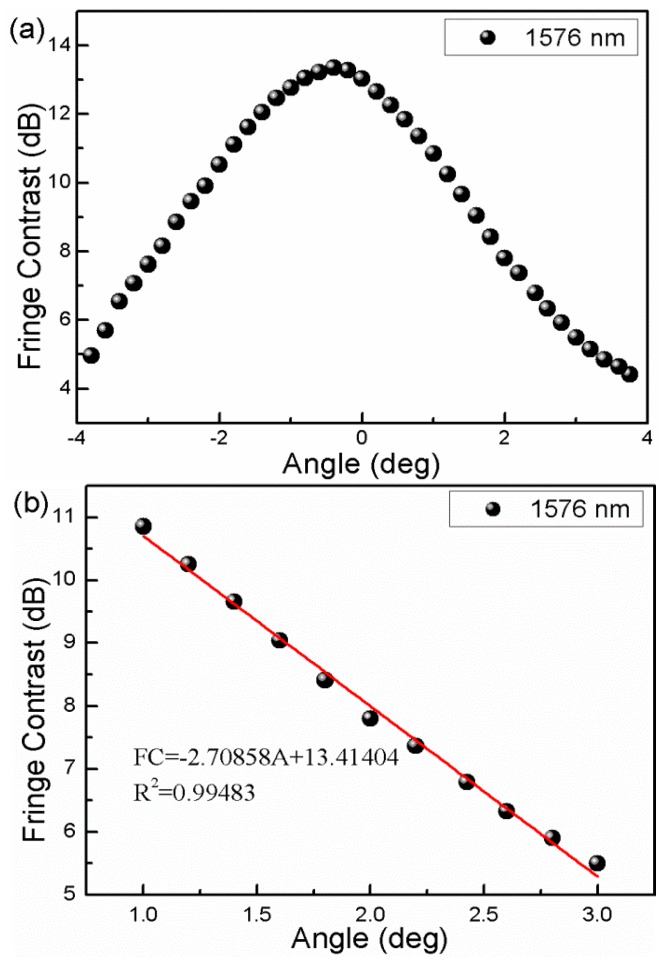

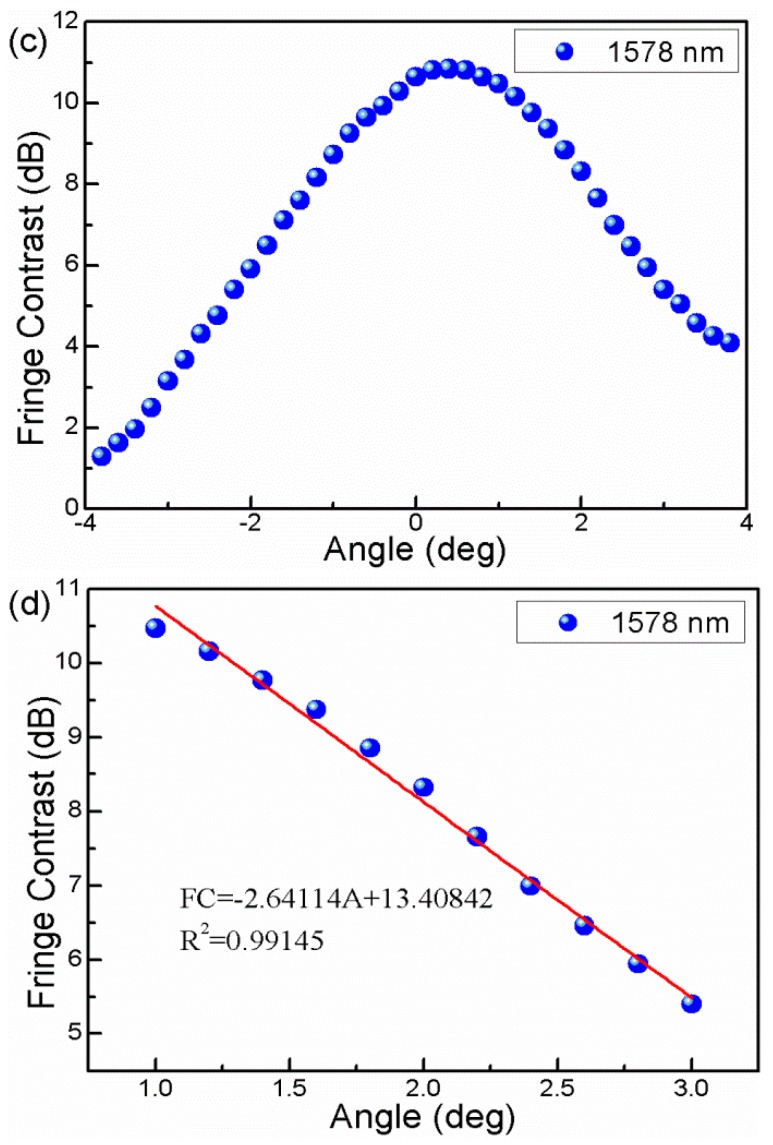
(**a**) Fringe contrast variation of interference valley near1576 nm; (**b**) Fringe contrast response linear fitting from 1° to 3° of 1576 nm valley; (**c**) Fringe contrast variation of interference valley near1578 nm; (**d**) Fringe contrast response linear fitting from 1° to 3° of 1578 nm valley.

**Figure 6:**
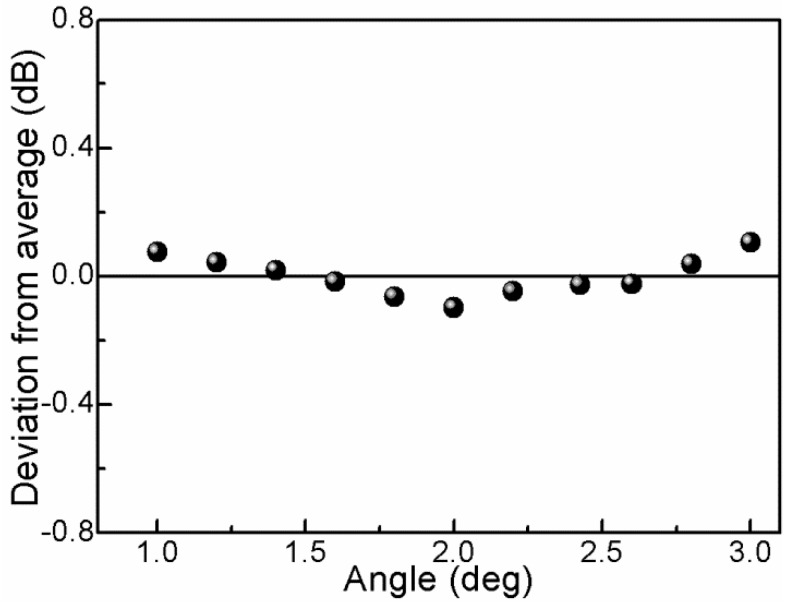
Fringe contrast fluctuation *versus* bending angle perturbation.

In order to characterize the bending response of the interferometer, the fiber is bent from 0° to ±4° using a rotator with a rotation resolution of 0.2°. We plot the fringe contrast as a function of the bending angles, as the blue points show in Figure 5a. It can be seen that, when the bending angle is varied ranging from 0° to ±1°, the fringe contrast of interference pattern responds strongly to the fiber-bending, which indicates that the coupling and recoupling of the core-to-cladding modes play a key role. With increasing the bending angle from 1° to ±3°, the bending-induced loss of the interferometer will mainly determine the light transmission, in which the TCF contributes to the bending loss. The fitting function of the fringe contrast *versus* bending angles can be written as I=−2.70858A+13.41404, as shown in Figure 5b.The error bars are presented in Figure 6, from which the standard deviation of the fringe contrast for the 11 instances of bending measurement can be calculated to be 0.059 dB, *i.e.*, the bending measurement accuracy can be written as (0.059/2.70858) deg. = 0.022 deg.

In addition, we characterize the bending angle responses of the fringe contrasts with the different wavelength of 1578 nm, as shown in Figure 5c. It can be seen that the bending angle sensitivity is different over the whole the angle range. For example, at the linear response range of 1° to 3°, the interferometer presents another angle sensitivity of −2.64 dB/deg., as shown in Figure 5d. This difference is attributed to that of the multi-order cladding modes’ interference, which is in agreement with the principle analysis above.

It is worth noting that the structure of the proposed fiber inclinometer is circularly symmetric, which indicates that the fringe contrast data points during the bending measurement should be distributed on both sides of the 0° point symmetrically. However, the results presented in Figure 5a,c do not agree with the expected result. This is attributed to the following two effects. One is that the experimental setup for characterizing the sensing response is not perfectly symmetric, which will create a bending radius difference at the same bending angle of the opposite direction. The other one is that some high-order cladding modes participate in the interference of the present asymmetric electric field profile, which shows a direction-dependent bending response.

**Figure 7:**
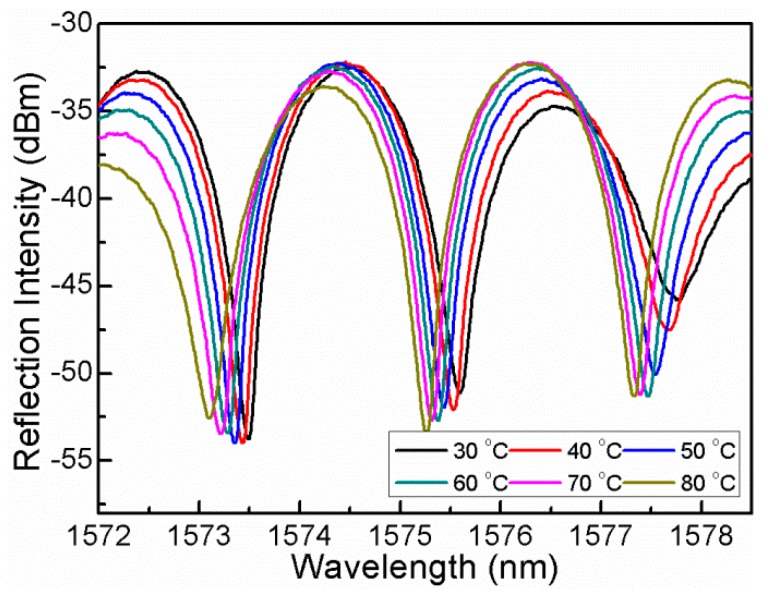
Interference spectral response to temperature increase from 30 °C to 80 °C.

In order to characterize its temperature response, the proposed sensor was placed into a temperature oven, which controls the temperature raises from 30 °C to 80 °C with a step of 10 °C.As shown in Figure 7, the interference spectrum presents a red-shift. However, it is unexpected that the spectral fringe contrast also changes indefinitely, which indicates that a cross-talk is induced by the temperature variations.

**Figure 8:**
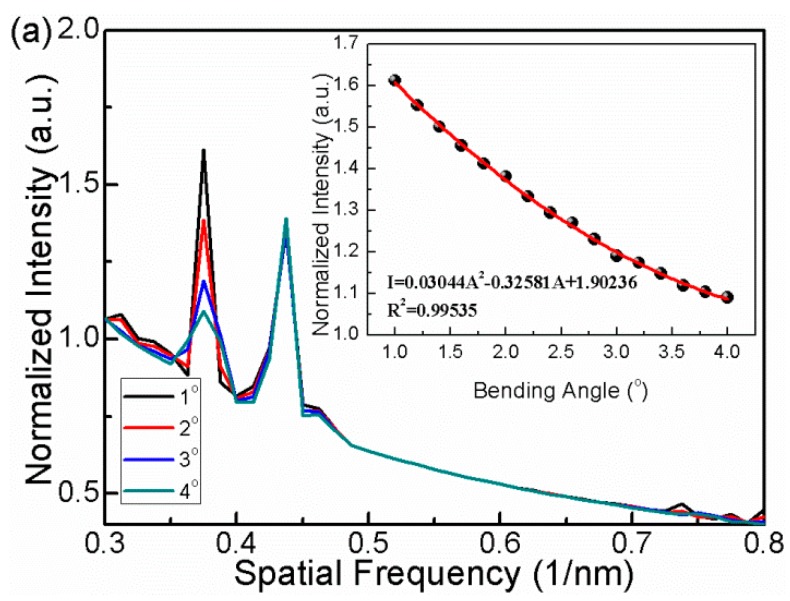

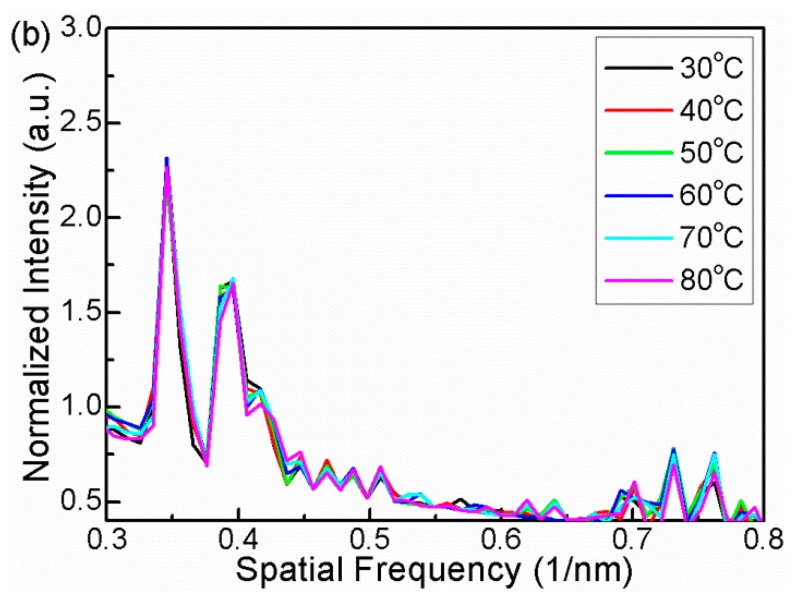
(**a**) FFT spectrum of the interference response to different bending angles. Insert is normalized intensity of “cladding mode 1” response to different bending angles; (**b**) FFT spectrum of the interference response to different temperatures.

A proposed solution to the cross-talk is to employ the spatial frequency for responding to the TCF bending change. As shown in Figure 8a, the intensity of cladding mode 1 significantly decreases with the increasing TCF bending. We plot the intensity as the function of bending angles, and a quadratic function relationship can be written as I = 0.03044A^2^ − 0.32581A + 1.90236. In Figure 8b, temperature changes do not influence the spatial frequency spectrum. Therefore, the cross-talk problem can be solved by interrogating the interference in the frequency domain.

## 4. Conclusions

In this paper, we propose a FFPI-based inclinometer. Experimental results and theoretical analysis show that, with the increase of the bending angle, the dominant factor of the spectrum’s variation changes from modes coupling to bend-induced loss of the interferometer, which exhibits bending sensitivity change. Compared with the previously reported sensors, the proposed inclinometer here presents some advantages, such as a compact size of smaller than 3 mm and a high tilt-angle sensitivity of 2.71 dB/deg. Especially, an intensity interrogation technique is employed for measuring the fiber bending angles based on the bending-induced coupling and recoupling loss of cladding modes, which can effectively eliminates the temperature-induced cross-talk. Moreover, it can provide remote sensing as a reflection probe, and the fabrication is simple and cost-effective, which makes it a good candidate for structural health monitoring.
